# Draft Genome Sequences of the Antarctic Endolithic Fungi *Rachicladosporium antarcticum* CCFEE 5527 and *Rachicladosporium* sp. CCFEE 5018

**DOI:** 10.1128/genomeA.00397-17

**Published:** 2017-07-06

**Authors:** Claudia Coleine, Sawyer Masonjones, Laura Selbmann, Laura Zucconi, Silvano Onofri, Claudia Pacelli, Jason E. Stajich

**Affiliations:** a Department of Ecological and Biological Sciences, University of Tuscia, Viterbo, Italy; b Department of Plant Pathology and Microbiology and Institute of Integrative Genome Biology, University of California, Riverside, Riverside, California, USA

## Abstract

The draft genome sequences of *Rachicladosporium antarcticum* CCFEE 5527 and *Rachicladosporium* sp. CCFEE 5018 are the first sequenced genomes from this genus, which comprises rock-inhabiting fungi. These endolithic strains were isolated from inside rocks collected from the Antarctic Peninsula and Battleship Promontory (McMurdo Dry Valleys), Antarctica, respectively.

## GENOME ANNOUNCEMENT

The past decade has revealed an unexpected fungal diversity associated with rocks, which serves as a primary substrate colonized by microorganisms in extreme dry and cold or hot environments. Under these harsh conditions, active growth is rare on exposed surfaces, and endolithism is a necessary ecological adaptation for survival (1). Black meristematic fungi are a morpho-ecological group of ascomycetes with a peculiar tendency to the extremes and are characterized by melanin pigmentation. They are typical and abundant members of Antarctic cryptoendolithic communities (2). These fungi are equally named black yeasts or microcolonial fungi and rock inhabitant fungi when found growing within rocks (3–7). We produced draft genome sequences of the Antarctic fungi *Rachicladosporium antarcticum* CCFEE 5527 Onofri & Egidi (2) and *Rachicladosporium* sp. strain CCFEE 5018 to provide genome resources to study fungal adaptation to extreme environments and endolithic lifestyles. These genomic resources may give clues for studying the evolution of extremophiles and stress adaptation in these enigmatic fungi.

*Rachicladosporium* strains were obtained from the Culture Collection of Fungi from Extreme Environments (Viterbo, Italy) and were cultured from inside Antarctic rocks. Species designation of *Rachicladosporium* sp. strain CCFEE 5018 is still being determined, and the internal transcribed spacer sequence is 98.6% identical to *Rachicladosporium monterosium* strain CBS 137178 Isola & Zucconi (2). Cultures were grown on 2% malt extract agar. DNA was extracted using a cetyltrimethylammonium bromide (CTAB)-based protocol (8). Two phenol-chloroform purification steps were used to eliminate melanin from the DNA. Total genomic DNA was sheared with a Covaris S220 ultrasonicator. Sequencing libraries were constructed using NeoPrep TruSeq Nano DNA sample prep (Illumina, Inc., San Diego, CA). Libraries were normalized, pooled, and sequenced on an Illumina MiSeq with 2 × 300 paired-end reads. *R. antarcticum* was sequenced to a depth of 41× and *Rachicladosporium* sp. CCFEE 5018 to 175× to improve assembly quality.

Genome assembly with MaSuRCA version 2.3.2 (9) was followed by vector sequence filtering with Sequin (https://www.ncbi.nlm.nih.gov/Sequin/). Redundant contigs which aligned by MUMMer (10) at 95% across their entire length were removed. The *R. antarcticum* genome assembly was 47.4 Mb (number of contigs, 267; *N*_50_, 896 kb; *L*_50_, 20). The initial *Rachicladosporium* sp. CCFEE 5018 assembly was fragmented (2,099 contigs) but was scaffolded by synteny to *R. antarcticum* with Satsuma2 (11) and Mercator (12) into 233 scaffolds (*N*_50_, 1.35 Mb; *L*_50_, 12). The genomes were annotated with Funannotate utilizing Augustus (13), GeneMark.hmm-ES (14), and EVM (15) and prepared for GenBank with Genome Annotation Generator (16). Gene function predictions were assigned by matches to the Pfam (17), MEROPS (18), CAZy (19), InterProScan (20), and Swiss-Prot databases (21). Product descriptions were transferred from homologs with 60% similar alignments across 60% of the protein length. A total of 18,781 protein-coding genes were predicted in *R. antarcticum* and 18,892 in *Rachicladosporium* sp. CCFEE 5018.

### Accession number(s).

These whole-genome shotgun projects have been deposited at DDBJ/ENA/GenBank under the accession numbers NAJO00000000 and NAEU00000000. The versions described in this paper are the first versions, NAJO01000000 and NAEU01000000.
